# Association between androgen receptor gene alteration and osteoporosis in Chinese Han elderly men

**DOI:** 10.7717/peerj.14782

**Published:** 2023-02-13

**Authors:** Xin Huang, Zhengdong Zhang, Liangxuan Zou, Wenbo Li, Jun He

**Affiliations:** 1 Collaborative Innovation Center of Sichuan for Elderly Care and Health, Chengdu Medical College, Sichuan, China; 2 Department of Orthopedics, Chengdu Seventh People’s Hospital (Orthopedics, Affiliated Cancer Hospital, Chengdu Medical College), Sichuan, China; 3 Department of Orthopedics, First Affiliated Hospital of Chengdu Medical College, Sichuan, China

**Keywords:** Chinese Han older men, Osteoporosis, Androgen receptor gene alteration, Blood glucose, Blood lipids

## Abstract

**Objective:**

To explore the role of blood glucose, blood lipids, and androgen receptor gene (CAG)n genotype in the pathogenesis of osteoporosis in Chinese Han men and to provide theoretical value for screening people susceptible to osteoporosis.

**Methods:**

Patients who visited the First Affiliated Hospital of Chengdu Medical College from February 2021 to October 2021 were selected as research subjects to measure bone density by double-energy X-ray, osteoporosis patients as osteoporosis group (40 patients), and non-osteoporosis patients as the control group (40 patients). The STR method detected the repeat times of the androgen receptor gene (CAG)n in the two groups. The repeat times ≤22 were the SS genotype, and >22 were the LL genotype. Meanwhile, the patient’s age, body mass index (BMI), blood glucose, blood lipids, calcium, phosphorus, and alkaline phosphatase examined on day one after admission were collected, and the statistical analysis was performed using SPSS 26.0.

**Results:**

The results of the univariate analysis showed that there was no significant difference in age, calcium, phosphorus, alkaline phosphatase, and glycosylated hemoglobin between the two groups (*P* > 0.05). There were significant differences in average blood glucose, total cholesterol, triglyceride, high-density lipoprotein, low-density lipoprotein, and genotype frequency (*P* < 0.05). The multivariate logistic regression analysis results showed significant differences in total cholesterol and genotype frequency between the two groups (*P* < 0.05).

**Conclusion:**

Androgen receptor LL genotype and elevated total cholesterol may be the risk factors for osteoporosis in older men of Han nationality.

## Introduction

Osteoporosis is a manifestation of the body’s natural decline as a result of aging. It is a systemic bone metabolic disease characterized by low bone mass and destruction of the bone tissue microstructure, resulting in increased bone brittleness and susceptibility to fracture. It has been estimated that it affects approximately 200 million women and men worldwide, mainly over 60 (Noh, Yang & Jung, 2020). With the advent of an aging society, older men with osteoporosis are not that uncommon. Rinonapoli et al. (2021) conducted a systematic review of the epidemiological characteristics of osteoporosis, indicating that osteoporosis is underestimated in both men and women, particularly in men.

Osteoporotic fractures are a significant health problem that affects a patient’s quality of life. It has been reported that 1/2 and 1/5 of all women and men experience brittle fractures at least once in their lifetime, respectively (Gregson et al., 2022). Studies have shown that the incidence of femoral neck fractures in male patients with osteoporosis is four times higher than that of female patients with osteoporosis (Shigehara et al., 2017). Patients with osteoporotic fractures are prone to postoperative pulmonary infections, venous thrombosis, and other serious complications that affect their quality of life. Therefore, not only should we pay attention to the bone mineral density of women, but of men as well. Early detection of male osteoporosis can play a key role in the minimization of serious physical and mental health consequences.

The occurrence of osteoporosis is closely related to diabetes, and its mechanism may be related to chronic hyperglycemia, increased of advanced glycation end products and oxidative stress (Mohsin et al., 2019). Disorder of lipid metabolism pathways affect osteocytes in varying degrees, leading to the development of osteoporosis (Yin, Li & Zhang, 2019). Therefore, we aimed to determine if indicators such as blood glucose levels and lipid profiles were risk factors for osteoporosis.

Androgen is essential in male bone growth, peak bone mass acquisition, and bone mass maintenance hormone present after puberty (Mohamad, Soelaiman & Chin, 2016). Chen et al. (2019) believed that androgen deficiency is closely related to bone loss and increased fracture risk. Androgens can regulate the function of osteoblasts and osteoclasts not only through the binding of androgen receptors (AR) on osteoclasts and osteoblasts but also by transforming into estrogen and regulating bone metabolism through estrogen receptors, in which AR plays a vital role (Khosla, 2015). Androgen receptors are expressed in the bone marrow mesenchymal cells (the source of osteoblasts), osteoblasts, osteocytes, and osteoclasts. Androgens promote osteogenesis and inhibit osteoclasts through AR. A copy of the gene encodes the androgen receptor on the X chromosome, which is composed of eight exons and seven introns. The first exon has a CAG repeat alteration, that ranges from 10–35, and there are ethnic differences in the repeat alteration (Edelsztein & Rey, 2019; Tirabassi et al., 2015).

The alteration of the AR gene may change the structure of the AR and affect the transcriptional activity of the androgen receptor gene (Zhou et al., 2016). The length of the long CAG repeat sequence was inversely proportional to the transcriptional activity of the AR gene (Ashraf et al., 2022). Androgens have a certain effect on osteoporosis through androgen receptors. However, the number of androgen receptor CAG repeats has an effect on the transcriptional activity of androgen receptor genes. Therefore, we explored the effects of the androgen receptor CAG repeat sequence on osteoporosis.

## Methods

### Selection criteria of patients

Forty patients with osteoporosis who received care from the First Affiliated Hospital of Chengdu Medical College between February 2021 to October 2021 were selected as the osteoporosis group, and 40 non-osteoporotic patients as the control group. The control group was fracture patients. All selected patients received written informed consent, and this study was approved by the Ethics Committee of Chengdu Seventh people’s Hospital. Inclusion criteria: ≥65 years old; local Han male whose parents are both of local Han nationality; no heart, liver, and other vital organ diseases. Exclusion criteria: patients who take drugs that affect bone metabolism; osteoporosis caused by rheumatoid arthritis, hyperthyroidism, tuberculosis of bone and joint, severe liver and kidney diseases; patients with incomplete clinical data; patients with internal fixation in lumbar vertebrae or bilateral hips.

### Bone mineral density measurement

The bone mineral density of the L1 to L4 vertebrae and unilateral hip was measured using dual-energy radiography. Patients with a *T* value ≤−2.5 were in the osteoporosis group, and those with a *T* value >−2.5 were in the control group.

### Blood glucose and blood lipids

After admission, patients were routinely examined for blood glucose (mean blood glucose, glycosylated hemoglobin) and blood lipids (total cholesterol, triglyceride, high-density lipoprotein, and low-density lipoprotein). The data was stored and can be accessed through the electronic medical record system. Calcium, phosphorus, and alkaline phosphatases levels were also measured and stored.

### Main reagents and instruments

dNTP, TaKaRa LA TaqTM polymerase (Chengdu Lilai Biotechnology Co., Ltd, Chengdu, China), Hi-Di deionized formamide, POP-7 Polymer, GS-500LIZ molecular internal standard (Shanghai Da Krypton Biotechnology Co., Ltd, Shanghai, China), Vortex oscillator (Jiangsu Haimen Qilin Medical instrument Factory, Jiangsu, China), PCR instrument (Esco Shanghai Trading Co., Ltd, Shanghai, China), Electrophoretic apparatus (Beijing Baygene Biotech Co., Ltd, Beijing, China), ABI3730xl Sequencer (Applied Biosystems Company, Waltham, MA, USA).

### Androgen receptor (CAG)n genotype

Patients were required to fast and a 5 ml venipuncture sample from the inside of the elbow was extracted, anticoagulated with ethylenediaminetetraacetic acid (EDTA) and stored at −80 °C until required for use. To analyze the sample, it was thawed at room temperature, DNA was extracted using a DNA extraction kit according to the manufacturer’s instructions. Fluorescent primers were synthesized, and androgen receptor CAG gene primers were designed and synthesized according to Zhang et al. (2013).

The PCR amplification of the DNA was performed using the fluorescent primers; forward: 5′-TCCAGAATCTGTTCCAGAGCGTGC-3′, and reverse: 5′-GCTGTGAAGGTTGCTGTTCCTCAT-3′.

The length of the amplified product was 222+3n. The PCR was performed using under the following conditions: 95 °C (10 min), one cycle. 95 °C (30 s), 62–52 °C (30 s), 72 °C (30 s), and ten cycles in sequence. 95 °C (30 s), 52 °C (30 s), 72 °C (30 s), and 25 cycles in sequence. 72 °C (7 min), one cycle. The PCR product was stored at 4 °C. The PCR fragments were separated through capillary electrophoresis using AB13730xl sequencer.

**Figure 1:**
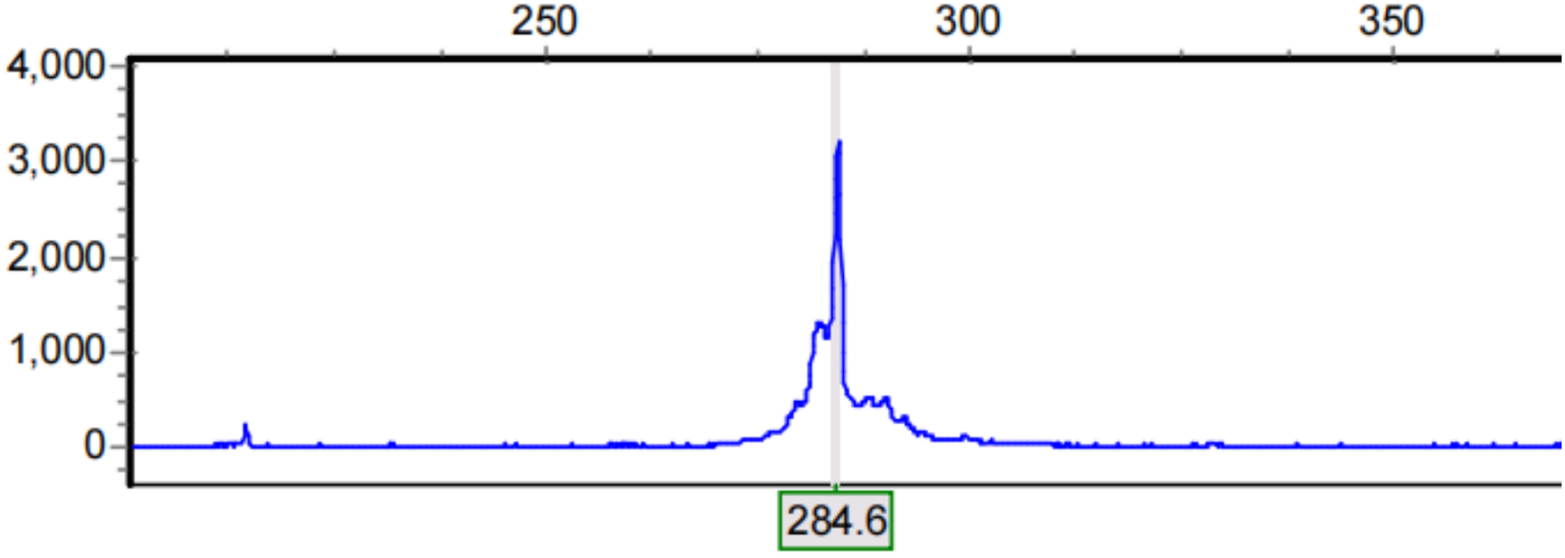
Results of capillary electrophoresis. Abscissa represents the size of capillary electrophoresis fragment and ordinate represents fluorescence signal intensity. The electrophoretic fragment with the highest fluorescence signal intensity was selected as the length of the amplification product.

The abscissa of the capillary electrophoresis peak map represents the size of the capillary electrophoresis fragment and the ordinate represents the fluorescence signal intensity (Fig. 1). Lastly, GeneMarker data were analyzed to determine the number of CAG repeats in each sample and this was inferred according to the formula *n* = (length of the amplified product-common length of 222 bp/3). Those with ≤22 repeats were the SS genotype, and those with >22 repeats were the LL genotype (Zhang et al., 2013; Li et al., 2017).

### Data analysis

The data was analyzed using statistical software SPSS 26.0. A *T*-test and Wilcoxon rank sum test were used for continuous variables. The classified variables are expressed as counts and percentages and analyzed by chi-square test. First, the influencing factors were analyzed by single factor analysis, and then the factors with statistical differences were analyzed by multi-factor logistic regression. Statistical significance was considered as *P* < 0.05.

## Results

### Effect of general condition of patients on osteoporosis

There were no significant differences (*P* > 0.05) in age, calcium, phosphorus, and alkaline phosphatase levels between the two groups, but there was a significant difference in BMI (*P* < 0.05) between the two groups, as shown in Table 1.

**Table 1:** Effect of general condition of patients on osteoporosis.

	Osteoporosis group	Control group	t	*P*
Age	77.20 ± 6.57	75.93 ± 6.65	0.863	0.391
BMI	21.26 ± 3.45	23.23 ± 3.04	2.724	0.008
Calcium (mmol/L)	2.19 ± 0.18	2.23 ± 0.16	1.107	0.272
Phosphorus (mmol/L)	1.19 ± 0.13	1.13 ± 0.19	1.488	0.141
Alkaline phosphatase (mmol/L)	97.93 ± 5.98	96.92 ± 6.54	0.721	0.473

**Note:**

There was no significant difference in age (*P* = 0.391), calcium (*P* = 0.272), phosphorus (*P* = 0.141), and alkaline phosphatase (*P* = 0.473) between the two groups, but there was a significant difference in BMI (*P* = 0.008) between the two groups.

### Effects of blood glucose and blood lipid on osteoporosis

Univariate statistical analysis was statistically significant (*P* < 0.05) for average blood glucose, glycosylated hemoglobin, cholesterol, triglyceride, high- and low-density lipoprotein levels between the two groups (Table 2).

**Table 2:** Effects of blood sugar and blood lipid on osteoporosis.

	Osteoporosis group	Control group	T/Z	*P*
Average blood glucose (mmol/L)	8.79 ± 2.10	7.30 ± 2.34	3.003	0.004
Glycosylated hemoglobin (%)	6.95 ± 1.81	6.07 ± 1.54	−1.953	0.051
Total cholesterol (mmol/L)	5.16 ± 1.47	4.06 ± 0.70	−3.637	0.000
Triglyceride (mmol/L)	1.26 ± 0.31	1.08 ± 0.24	−3.547	0.000
High-density lipoprotein (mmol/L)	0.81 ± 0.13	0.74 ± 0.13	−2.473	0.013
Low-density lipoprotein (mmol/L)	1.63 ± 0.42	1.49 ± 0.19	−2.667	0.008

**Note:**

Univariate statistical analysis was carried out on the average blood glucose (*P* = 0.004), glycosylated hemoglobin (*P* = 0.022), total cholesterol (*P* = 0.000), triglyceride (*P* = 0.004), high density lipoprotein (*P* = 0.026) and low-density lipoprotein (*P* = 0.043) between the two groups. There were statistical differences in blood glucose and lipids between the two groups.

### Effect of androgen receptor (CAG)n genotype on osteoporosis

The SS and LL genotype frequency of osteoporosis and the control was 52.5% and 85%; and 47.5%, and 15%, respectively. The two groups were statistically significant (*P* < 0.05), as shown in Table 3.

**Table 3:** Effect of androgen receptor (CAG)n genotype on osteoporosis.

	SS genotype	LL genotype
Osteoporosis group (percentage)	21 (52.5%)	19 (47.5%)
Control group (percentage)	34 (85%)	6 (15%)
χ²	9.833	
*P*	0.002	

**Note:**

The SS genotype frequency of osteoporosis was 52.5%, LL 47.5%, control SS 85%, and LL 15%, and was statistically different between the two groups with a *p*-value of 0.002.

### Multivariate logistic regression analysis of the influencing factors of osteoporosis

The influencing factors with statistical differences were selected for logistic regression analysis. The results suggested that the factors affecting bone mineral density in older men of Han nationality were total cholesterol (*P* = 0.048) and androgen receptor (CAG)n genotypes (*P* = 0.012). The risk of osteoporosis in elderly Han men with the LL genotype was 5.6 times higher than that in patients with the SS genotype, and each unit increase in cholesterol increased the risk of osteoporosis by 2.056 times, as shown in Table 4.

**Table 4:** Multivariate logistic regression analysis of the influencing factors of osteoporosis.

	Regression coefficient	Standard error of regression coefficient	OR	*P*
Total cholesterol	0.721	0.364	2.056	0.048
Genotype (LL)	1.725	0.688	5.612	0.012

**Note:**

The influencing factors with statistical differences were selected for Logistic regression analysis. The results suggested that the factors affecting bone mineral density in older men of Han nationality were total cholesterol (*P* = 0.048) and androgen receptor (CAG)n genotypes (*P* = 0.012). The risk of osteoporosis in elderly Han men with LL genotype was 5.6 times higher than that in patients with SS genotype, and each unit increase in cholesterol increased the risk of osteoporosis by 2.056 times.

## Discussion

Osteoporosis is a chronic disease in the elderly, and its pathogenesis is closely related to the genes (Lei et al., 2006). The relationship between estrogen receptor genes and osteoporosis has been widely studied (Zhu et al., 2018). Some scholars have confirmed that the susceptibility of Chinese Han women to osteoporosis may be affected by ER-α *PVU*-II and ER-β Alu-I gene alterations, and that women with An and P alleles have a higher risk of osteoporosis (Xiang, He & Jiang, 2018). Although the prevalence of osteoporosis in men is lower than that in women, with the advent of an aging society, the number of older people is increasing, and the number of men with osteoporosis should not be underestimated. Moreover, the role of androgen receptor genotypes in the pathogenesis of osteoporosis is unclear; therefore, this study discusses the relationship between androgen receptor CAG gene alteration and osteoporosis. The results of the multivariate logistic regression analysis in this study suggest that the LL genotype is related to susceptibility to osteoporosis in elderly Han men.

In this study, we focused not only on the role of genes in the pathogenesis of osteoporosis but also on the influence of factors such as patient age, BMI, blood glucose, and blood lipids on bone mineral density. Sun et al. (2020) showed that BMD was positively correlated with BMI at 18.0–31.2 kg/m^2^ and BMI from 31.3–40.6 kg/m^2^ was negatively associated. This is generally in line with the conclusion suggested by Crivelli et al. (2021) as it was indicated that a BMI of ≥40 kg/m^2^ increases the risk of lumbar fractures. The dual effect of BMI on BMD may be due to multiple mechanisms such as weight, bone formation, and the bone marrow microenvironment (Sun et al., 2020). Stress hyperglycemia in relation to an admission disease, *e.g*., bone fracture, can produce errors in the blood glucose data. Therefore, this study reflects the blood glucose levels by obtaining the patient’s average blood glucose and glycated hemoglobin levels. Hyperglycemia affects bone mineral density and it is suggested that hyperglycemia increases the levels of inflammatory cytokines and glycosylated end products such as interleukin six and tumor necrosis factor, which can trigger apoptosis and weaken the activity of osteoblasts (Araújo et al., 2022). In addition, hyperglycemia can promote osteoclast differentiation and bone resorption by increasing the expression levels of factors, such as RANK and RANKL (Wang et al., 2016; Qu et al., 2018).

Several studies have shown that triglycerides, total cholesterol, low-density lipoprotein, and BMD may be inversely correlated (Chen et al., 2018; Yang et al., 2019; Li et al., 2015). Although high concentrations of high-density lipoprotein levels were more protective against cardiovascular and cerebrovascular vessels, Cui et al. (2016) confirmed that high-density lipoprotein above 1.56 mmol/L was a risk factor for osteoporosis. Low concentrations of high-density lipoprotein inhibit osteoblast differentiation and function, reducing bone mass, therefore, suggesting that high-density lipoprotein is a protective factor responsible for osteoporosis (Papachristou et al., 2017). High cholesterol concentrations could have an effect on bone density, as this reduces the amount of oxysterol in the surrounding tissues, thus reducing Mesenchymal stem cell differentiation into osteoblasts (Li et al., 2015).

Although we observed that BMI, blood glucose, blood lipids, and genotypes all played a role in the pathogenesis of osteoporosis in older adults of Han nationality in the univariate analysis, only the effects of genotypes and total cholesterol on osteoporosis were observed in the multivariate logistic regression analysis. In addition, this study also discussed routine examination indexes, such as serum calcium, serum phosphorus, and alkaline phosphatase, which play a specific role in bone formation and metabolism. The serological detection of bone metabolic indexes has the advantages of a simple, convenient, and short reporting cycle; therefore, we investigated whether there is a correlation between serum calcium, phosphorus, alkaline phosphatase, and male osteoporosis. However, there were no relevant conclusions in our study.

This study has some limitations. First, the sample size of this study was small, which can be further expanded to explore the effects of BMI, blood glucose level, and other factors on osteoporosis in elderly Han men. Second, this study only included older adults of Han nationality in China and did not explore other races. Finally, this study only discussed the effect on osteoporosis in terms of genotypes, and the specific mechanism of androgen receptor on bone mineral density needs to be further studied.

In summary, androgen receptor gene alteration and total cholesterol are associated with osteoporosis in elderly Han men. LL genotype and high cholesterol are risk factors for osteoporosis in older adults of Han nationality, but we did not observe the effects of BMI, blood glucose, calcium, phosphorus, and alkaline phosphatase on osteoporosis.

## Supplemental Information

10.7717/peerj.14782/supp-1 Supplemental Information 1 The patient’s general condition, blood lipids, blood sugar, (CAG) repeat times and other data.

## Additional Information and Declarations

### Data Availability

The following information was supplied regarding data availability: The raw data are available in the Supplemental Files.
